# [Retracted] MicroRNA-375 inhibits esophageal squamous cell carcinoma proliferation through direct targeting of SP1

**DOI:** 10.3892/etm.2025.12990

**Published:** 2025-10-06

**Authors:** Hui Xu, Jialong Jiang, Jingjun Zhang, Liang Cheng, Song Pan, Yuanhai Li

Exp Ther Med 17:1509–1516, 2019; DOI: 10.3892/etm.2018.7106

Following the publication of this paper, it was drawn to the Editor’s attention by a concerned reader that certain of the colony formation assay featured in Fig. 2C and E on p. 1512 appeared to have been duplicated, where different experimental conditions were meant to have applied. Upon performing an independent analysis of the data in the Editorial Office, it came to light that some of the same data had already been submitted for publication in an article written by different authors at different research institutes to *Journal of Experimental & Clinical Cancer Research*.

In view of the fact that the abovementioned data had already apparently been published previously, the Editor of *Experimental and Therapeutic Medicine* has decided that this paper should be retracted from the Journal. The authors were asked for an explanation to account for these concerns, but the Editorial Office did not receive a reply. The Editor apologizes to the readership for any inconvenience caused.

